# INNOVATIVE TECHNOLOGIES: MRI-Based Atlas of the Developing Mouse Brain Debuts

**DOI:** 10.1289/ehp.119-a165

**Published:** 2011-04

**Authors:** David C. Holzman

**Affiliations:** **David C. Holzman** writes on science, medicine, energy, economics, and cars from Lexington and Wellfleet, MA. His work has appeared in *Smithsonian*, *The Atlantic Monthly*, and the *Journal of the National Cancer Institute*

The mouse is one of the major animal models for toxicologic research, but histologic analyses are notoriously slow, taking a week or longer. A new magnetic resonance imaging (MRI)–based atlas of the developing mouse brain now provides a badly needed baseline for studies of how pollutants and genetic mutations affect brain development in mouse models.^1^ The atlas traces the development and growth not only of the entire mouse brain and its constituent parts but also of its white matter and connectivity,^2^ day by day, from embryonic day 12 to postnatal day 80.

The new brain atlas greatly reduces the amount of work necessary to determine the effect of either mutations or pollutants on brain development, says coauthor Susumu Mori, a professor in the Department of Radiology at the Johns Hopkins University School of Medicine. Typically, one would harvest tissue samples at different points in development and perform histologic analyses, he says, but the lack of prior knowledge of which structure is altered by a given exposure necessitates creating hundreds of histology sections—an arduous task. “Our idea,” he says, “is to do three-dimensional microimaging [with MRI], which can capture the anatomy of the entire brain within a day.” For that, the mouse brain atlas provides the baseline.

This baseline is ideal for determining changes in growth rates that might arise from neurotoxicities, says G. Allan Johnson, director of the Duke University Center for *in Vivo* Microscopy, who was not involved in the research. “My personal opinion,” he says, “is that MR imaging—MR histology in particular—will become one of the major ways to produce quantitative measures of environmental toxicology.”

The atlas quantifies the whole brain of the widely used C57BL/6 mouse, as well as the neocortex, cerebellum, hippocampus, and more than 17 other substructures, with additional substructures being added steadily. It also maps the white matter tracts and the gray matter structures. Additionally, it characterizes anatomical variability at several developmental stages.^1^ Complementary use of the high-resolution technique known as diffusion tensor imaging helped boost the normally poor tissue contrast provided in immature mouse brain samples by MRI alone.

“The ability to have a three-dimensional map of the developing brain is very important for studying neurotoxicology and developmental neuroscience,” says Tomás Guilarte, the Leon Hess professor of Environmental Health Sciences at Columbia University’s Mailman School of Public Health, who has worked in the past with Mori. In terms of future utility, Guilarte sees the mouse brain atlas as having a breadth of applications comparable to polymerase chain reaction.

The new atlas supercedes histology-based atlases that have very limited coverage of different developmental stages, says Mark Henkelman, director of the Mouse Imaging Center at the Hospital for Sick Children in Toronto, who was not involved in the research. Henkelman says the developmental breadth of the atlas could offer critical clues to predict whether a toxicant poses a special threat to pregnant mothers or children need to avoid, or whether it’s likely to affect the entire population.^3^
